# Diagnostic Performance of Multimodal Artificial Intelligence in Differentiating Ocular Surface Squamous Neoplasia from Benign Lesions: A Retrospective Diagnostic Accuracy Study

**DOI:** 10.3390/diagnostics16213475

**Published:** 2026-10-24

**Authors:** Laura De Luca, Feliciana Menna, Stefano Lupo, Enzo Maria Vingolo, Matteo Mario Carlà, Antonio Baldascino, Alessandro Meduri

**Affiliations:** 1 Ophthalmology Clinic, Department of Biomedical Sciences, University of Messina, 98122 Messina, Italy; laura.deluca21@gmail.com (L.D.L.);; 2 U.O.C. Ophthalmology, Department of Medical-Surgical Sciences and Biotechnologies, Sapienza University of Rome, Via Firenze 1, 04019 Terracina, Italy; 3 Ophthalmology Unit, Fondazione Policlinico Universitario A. Gemelli IRCCS, 00168 Rome, Italy

**Keywords:** ocular surface squamous neoplasia, conjunctival intraepithelial neoplasia, artificial intelligence, large language models, multimodal imaging, diagnostic accuracy, anterior segment optical coherence tomography, in vivo confocal microscopy

## Abstract

**Background/Objectives**: Ocular surface squamous neoplasia (OSSN), comprising conjunctival intraepithelial neoplasia (CIN) and invasive squamous cell carcinoma (SCC), can be difficult to distinguish from benign ocular surface lesions. We evaluated the diagnostic performance of a multimodal vision-language model in differentiating OSSN from selected benign lesions using standardized clinical and imaging inputs. **Methods**: In this retrospective diagnostic accuracy study, 90 histopathologically confirmed lesions (20 CIN, 20 invasive SCC, 20 pterygia, 20 pseudopterygia, and 10 conjunctival papillomas) from a single tertiary center were evaluated with ChatGPT-4o (OpenAI) under three predefined conditions: expert-structured text, image-only, and combined multimodal input. The model was required to select a single diagnosis from predefined categories. Histopathology was the reference standard; agreement with expert clinical consensus was assessed separately. Scenario authors and consensus raters were blinded to histopathology. **Results**: In the multimodal condition, diagnostic accuracy was 78.9% (71/90; 95% CI, 69.4–86.0%), sensitivity 82.5% (95% CI, 68.1–91.3%), specificity 76.0% (95% CI, 62.6–85.7%), positive predictive value 73.3%, negative predictive value 84.4%, positive likelihood ratio 3.44 and negative likelihood ratio 0.23; false-negative and false-positive rates were 17.5% and 24.0%. Agreement with expert consensus was moderate (κ = 0.58). Accuracy was 60.0% (54/90; 95% CI, 49.7–69.5%) with expert-structured text alone and 70.0% (63/90; 95% CI, 59.9–78.5%) with images alone; because case-level paired predictions were not retained, between-condition differences were assessed descriptively only. **Conclusions**: In this single-center, diagnostically enriched cohort, a multimodal vision-language model showed measurable but imperfect diagnostic performance under standardized experimental conditions. These exploratory findings do not support autonomous clinical use; prospective external validation across broader lesion spectra and real-world clinical pathways is required.

## 1. Introduction

Ocular surface squamous neoplasia (OSSN) is the most common non-pigmented malignancy of the ocular surface and includes a spectrum ranging from conjunctival intraepithelial neoplasia to invasive squamous cell carcinoma [1,2]. In the present study, OSSN is used as an umbrella term encompassing both pre-invasive conjunctival intraepithelial neoplasia (CIN) and invasive squamous cell carcinoma (SCC). CIN and invasive SCC are reported separately when subtype-specific diagnostic performance is considered. Early recognition is essential, as delayed diagnosis may allow tumor progression, whereas misclassification of benign lesions can expose patients to unnecessary surgical procedures and topical chemotherapeutic agents [3]. However, distinguishing malignant lesions from benign ocular surface conditions remains clinically challenging [4].

Several benign entities, including pterygium, pseudopterygium, and conjunctival papilloma, may demonstrate overlapping clinical features such as vascularization, limbal involvement, and surface irregularity [5,6]. Although slit-lamp examination is fundamental for initial assessment, adjunctive imaging modalities such as anterior segment optical coherence tomography (AS-OCT) and in vivo confocal microscopy (IVCM) have improved lesion characterization [7,8]. Nevertheless, diagnostic uncertainty persists, particularly in early or atypical presentations [9].

Artificial intelligence has shown growing utility in ophthalmology, particularly in image-based diagnostics [10]. Recent advances in vision-language models allow simultaneous processing of clinical and imaging data, potentially supporting structured diagnostic assessment [11]. Deep learning classifiers trained on slit-lamp photographs or in vivo confocal microscopy images have recently achieved high discrimination of OSSN and other ocular surface tumors, although performance for rarer entities remains limited [12,13]. Most recently, a domain-specific foundation model pretrained on approximately 0.76 million unlabeled ocular surface images and fine-tuned on histopathologically labeled slit-lamp and digital photographs achieved AUC values of 0.89–0.99 for distinguishing malignant, premalignant, and benign ocular surface tumors across internal, external, and prospective test sets, performing comparably to senior ophthalmologists [14]. General-purpose multimodal models such as GPT-4V and GPT-4o have been evaluated on ophthalmic images with variable, often moderate, accuracy [15,16,17]. Reasoning-oriented large language models, which generate explicit intermediate reasoning steps, have recently been shown to approach human-like clinical reasoning in ophthalmic question answering, reaching accuracies of approximately 90% on text-based multiple-choice questions [18]; such benchmarks, however, do not involve image interpretation or lesion-level diagnosis. Preliminary reports suggest that general-purpose models can generate plausible diagnostic and management hypotheses in ocular oncology and ocular surface tumor cases [19,20]. This approach may be particularly relevant in ocular surface oncology, where clinical decision-making typically relies on integrating multiple sources of information rather than a single modality [7,8].

In a previous study from our group, ChatGPT-4 correctly classified 85% of CIN, 75% of pterygia, and 70% of pseudopterygia using structured text-only summaries of clinical, AS-OCT, and IVCM findings, without processing clinical images [21]. Whether the addition of image input, and the extension of the diagnostic spectrum to invasive SCC and conjunctival papilloma, affects the diagnostic performance of multimodal AI systems in ocular surface lesions remains insufficiently investigated.

The purpose of this study was to evaluate the diagnostic performance of a multimodal vision-language artificial intelligence system in differentiating OSSN from selected benign ocular surface lesions under standardized expert-structured text, image-only, and combined multimodal input conditions. Secondary objectives included characterization of diagnostic errors and evaluation of performance across clinically relevant lesion subtypes.

## 2. Materials and Methods

### 2.1. Study Design and Setting

This retrospective diagnostic accuracy study was conducted at the Ophthalmology Clinic of the University Hospital of Messina, a tertiary referral center for ocular surface disease. Clinical records and imaging data from patients presenting with ocular surface lesions between January 2021 and October 2025 were reviewed. The study protocol was reviewed by the Ethics Committee of the University of Messina, which determined that formal ethical approval was not required because of the retrospective design and the use of anonymized data (reference No. 278/2021, dated 1 April 2021). The study was conducted in accordance with the Declaration of Helsinki. Reporting follows the STARD 2015 statement for diagnostic accuracy studies [22], with additional reference to the TRIPOD-LLM guideline for studies evaluating large language models [23]. In line with TRIPOD-LLM, the verbatim prompts, the dates of evaluation, the model version, and the interface settings are reported in Section 2.5 and in Supplementary Material S1.

### 2.2. Participants

Lesions were classified according to histopathological diagnosis as neoplastic or benign. Neoplastic lesions belonged to the ocular surface squamous neoplasia (OSSN) spectrum and comprised conjunctival intraepithelial neoplasia (CIN; n = 20), representing pre-invasive OSSN, and invasive squamous cell carcinoma (SCC; n = 20). Benign lesions comprised pterygium (n = 20), pseudopterygium (n = 20), and conjunctival papilloma (n = 10). Throughout the manuscript, OSSN is used as an umbrella term encompassing both CIN and invasive SCC. Available clinical and morphological characteristics were retrospectively extracted from the clinical records and imaging archive. These included lesion morphology, presence or absence of clinically visible keratin, lesion dimensions, limbal involvement expressed in clock hours, disease stage for OSSN lesions, and availability of AS-OCT and IVCM imaging. These variables were used, where available, for descriptive characterization and exploratory error analyses. None of the lesions included in the present study had been included in our previous text-only evaluation of ChatGPT-4 [21]; the two study cohorts comprised distinct patients.

### 2.3. Reference Standard

Histopathological diagnosis was considered the primary reference standard for diagnostic-performance analyses. Histopathological classifications were retrieved from the original pathology reports and were independent of AI-generated predictions.

Clinical expert assessment was considered separately from the histopathological reference standard. A clinical consensus assessment was established by three ocular surface specialists (LDL, FM, AM) based on the available clinical and imaging information. The same three specialists also wrote the expert-structured text scenarios (Section 2.4). Both the scenarios and the consensus assessment were produced while blinded to the histopathological diagnosis: the specialists worked exclusively from the de-identified clinical records and images, and the histopathological results were retrieved from the pathology reports and linked to each case only after scenario development and consensus assessment had been completed. Agreement with histopathology and agreement with clinical expert assessment were considered conceptually distinct outcomes.

### 2.4. Expert-Structured Text Scenario Development

Standardized expert-structured scenarios were developed for each patient using a predefined template, with terminology and structure standardized across cases. The predefined scenario template included the following standardized domains: demographic characteristics; relevant risk factors; symptom history; lesion location; slit-lamp appearance; vascular pattern; surface morphology; lesion mobility; and, when available, expert-derived textual descriptions of AS-OCT and IVCM findings. The same sequence and structure of information were maintained across cases. Scenario authors were blinded to the histopathological diagnosis at the time of writing (Section 2.3).

Because AS-OCT and IVCM findings were represented through expert-derived textual descriptors, the text-based condition already contained information originating from the imaging modalities. Accordingly, the text-based and multimodal conditions were not fully information-independent. Comparisons between these conditions should therefore be interpreted as comparisons between different representations and combinations of information rather than as estimates of the independent incremental diagnostic value of imaging.

### 2.5. Artificial Intelligence Model and Evaluation Protocol

A proprietary multimodal vision-language model (ChatGPT-4o, OpenAI, San Francisco, CA, USA) was evaluated through its official user interface. No fine-tuning, retrieval augmentation, external tools, architectural modifications, or custom code were used. All evaluations were performed during the study evaluation period using the GPT-4o model as made available through the ChatGPT web interface under a ChatGPT Plus subscription. The consumer interface did not expose the underlying model snapshot identifier or allow direct modification of decoding parameters such as temperature; therefore, provider-default settings were applied throughout. Each case was submitted in a newly opened, independent chat session, without access to previous conversations. The Memory function and custom instructions were disabled, and no web browsing, code execution, or other optional tools were used during the evaluations.

Each case was presented in an independent session using standardized instructions. No conversational history from previous cases was available, and no iterative prompting, correction, or feedback was permitted. For each evaluation, the model was instructed to select a single most likely diagnosis exclusively from the predefined diagnostic categories (CIN, invasive SCC, pterygium, pseudopterygium, or conjunctival papilloma) and to provide a corresponding management recommendation. The instruction was identical across cases within each condition and differed between conditions only in the description of the material provided (expert-structured text, images, or both).

Because the evaluated model is proprietary and may undergo provider-side updates, the results represent model behavior at the time of evaluation and exact reproducibility with subsequent model versions cannot be assumed. Each case was evaluated under three predefined conditions: (1) expert-structured text input, (2) image-only input, and (3) combined multimodal input. Cases were presented in randomized order, and each evaluation was conducted in an independent session. In the image-only and multimodal conditions, the de-identified slit-lamp photograph and, when available, representative AS-OCT and IVCM images were uploaded together with the instruction. Each case–condition combination was evaluated once; repeated runs of identical inputs were not performed.

Model outputs were recorded at the time of evaluation on condition-specific scoring sheets in which the diagnosis selected by the model for each lesion was marked as correct or incorrect against histopathology and tallied by histopathological category. These sheets provide, for each input condition, the aggregate 2 × 2 classification counts reported in Section 3. However, the scoring sheets were not linked by a persistent case identifier across conditions, and the full chat transcripts, including the free-text management recommendations, were not exported or archived, because the original analysis plan was limited to aggregate per-condition diagnostic accuracy and did not anticipate paired between-condition comparisons, repeated-run reproducibility analyses, or quantitative analysis of management recommendations. Consequently, case-level paired predictions and case-level management recommendations could not be retrieved for retrospective reanalysis. We acknowledge this as a limitation of the original protocol (Section 4).

### 2.6. Outcome Measures

The primary outcome was diagnostic accuracy for differentiating OSSN from benign lesions relative to histopathology. Secondary diagnostic outcomes included sensitivity, specificity, positive predictive value (PPV), negative predictive value (NPV), positive likelihood ratio (LR+), negative likelihood ratio (LR−), false-positive rate, and false-negative rate.

Additional outcomes included lesion-specific diagnostic accuracy, agreement with the reference standard, management recommendations, and descriptive characterization of diagnostic errors according to available clinical and morphological lesion features.

Because the prespecified model output consisted of a single categorical diagnosis rather than a continuous or calibrated probability of neoplasia, each input condition yields exactly one operating point in receiver operating characteristic (ROC) space, defined by its sensitivity and false-positive rate (1 − specificity) after binary coding of the output (benign = 0; neoplastic = 1). These operating points are displayed graphically for descriptive purposes only. Conventional threshold-dependent ROC curves and area under the curve (AUC) values are not applicable to binary outputs and are therefore not reported.

### 2.7. Statistical Analysis

Diagnostic accuracy, sensitivity, specificity, PPV, NPV, LR+, and LR− were calculated with corresponding 95% confidence intervals. Ninety-five percent CIs for proportions were calculated with the Wilson score method, and CIs for likelihood ratios with the log method. Confusion matrices were constructed to summarize binary classification performance. All metrics were calculated separately for each of the three input conditions from the aggregate per-condition classification counts.

Because predictions under the different input conditions were generated on the same lesions, comparisons between input conditions require paired statistical methods. Because case-level paired predictions were not retained (Section 2.5), paired inferential comparisons (e.g., McNemar or Cochran’s Q tests) could not be performed, and no inferential comparison between input conditions is reported. Between-condition findings are therefore presented descriptively and are not interpreted as evidence of statistical superiority.

Agreement between AI classification and the reference standard was assessed using Cohen’s kappa. Exploratory descriptive analyses of diagnostic errors were performed according to available lesion characteristics.

All statistical analyses were performed using IBM SPSS Statistics, 2023 release (IBM Corp., Armonk, NY, USA).

### 2.8. AI Transparency

Generative artificial intelligence was used exclusively to generate diagnostic and management outputs from predefined study inputs and was not used to establish the histopathological reference diagnosis. All patient information submitted to the AI system was de-identified before evaluation, and no direct patient identifiers were included in the textual inputs or images.

No custom code or model fine-tuning was used. Clinical and imaging data were handled in accordance with applicable institutional patient-privacy and data-governance requirements. Because the evaluated model is proprietary, its architecture, weights, stochastic behavior, and provider-side updates were outside investigator control, limiting exact reproducibility.

## 3. Results

### 3.1. Study Population

A total of 90 patients with histopathologically confirmed ocular surface lesions were included. The cohort comprised 40 lesions within the OSSN spectrum (44.4%), including 20 cases of CIN and 20 invasive SCCs, and 50 benign lesions (55.6%), including 20 pterygia, 20 pseudopterygia, and 10 conjunctival papillomas. Baseline demographic and available clinical characteristics are summarized in Table 1.

### 3.2. Overall Diagnostic Performance

In the multimodal condition, 71 of 90 lesions were correctly classified, corresponding to an overall diagnostic accuracy of 78.9% (95% CI, 69.4–86.0%). Among the 40 histopathologically confirmed OSSN lesions, 33 were correctly classified, yielding a sensitivity of 82.5% (95% CI, 68.1–91.3%). Among the 50 benign lesions, 38 were correctly identified, corresponding to a specificity of 76.0% (95% CI, 62.6–85.7%). PPV was 73.3% (95% CI, 59.0–84.0%), and NPV was 84.4% (95% CI, 71.2–92.3%). LR+ was 3.44 (95% CI, 2.06–5.74), whereas LR− was 0.23 (95% CI, 0.12–0.46). The false-negative and false-positive rates were 17.5% and 24.0%, respectively (Table 2).

### 3.3. Performance Across Evaluation Modalities

**Figure 1:**
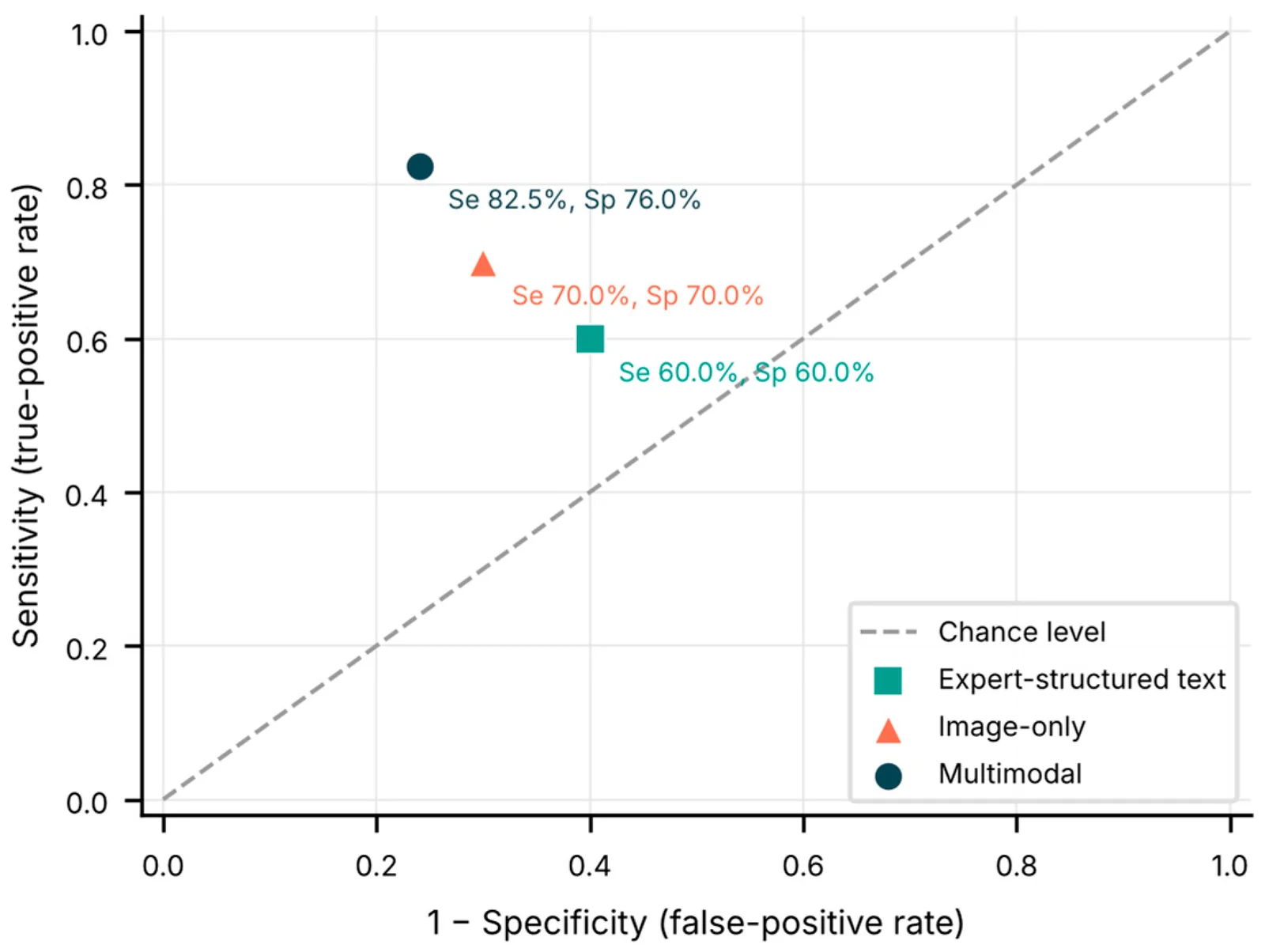
Operating points of the vision-language model in receiver operating characteristic (ROC) space under the three input conditions (expert-structured text, image-only, and multimodal). Because the model output was a single categorical diagnosis, each condition yields a single point defined by its sensitivity and false-positive rate (1 − specificity); no ROC curve or area under the curve is derived. Se, sensitivity; Sp, specificity. The dashed diagonal line represents chance-level classification.

Full diagnostic performance metrics for the three input conditions are reported in Table 3, and the corresponding operating points in ROC space are shown in Figure 1. With expert-structured text alone, 54 of 90 lesions were correctly classified (accuracy 60.0%; 95% CI, 49.7–69.5%), with a sensitivity of 60.0% (24/40; 95% CI, 44.6–73.7%) and a specificity of 60.0% (30/50; 95% CI, 46.2–72.4%). With images alone, 63 of 90 lesions were correctly classified (accuracy 70.0%; 95% CI, 59.9–78.5%), with a sensitivity of 70.0% (28/40; 95% CI, 54.6–81.9%) and a specificity of 70.0% (35/50; 95% CI, 56.2–80.9%). In the combined multimodal condition, accuracy was 78.9%, sensitivity 82.5%, and specificity 76.0% (Table 2). Correspondingly, the number of missed OSSN lesions was 16 with text alone, 12 with images alone, and 7 in the multimodal condition, and the number of benign lesions classified as neoplastic was 20, 15, and 12, respectively. Because predictions were generated on the same lesions, valid inferential comparisons would require paired case-level analyses; because case-level paired predictions were not retained (Section 2.5), these between-condition differences are presented descriptively and are not interpreted as evidence of statistical superiority. Qualitatively, misclassifications under the image-only condition were noted particularly in lesions with inflammatory or fibrovascular characteristics, whereas the multimodal condition showed fewer observed misclassifications in diagnostically ambiguous cases.

### 3.4. Performance by Lesion Subtype

Diagnostic accuracy was 85.0% for CIN and 80.0% for invasive SCC. Among benign lesions, accuracy was 75.0% for pterygium and 70.0% for pseudopterygium. A separate accuracy estimate is not reported for conjunctival papilloma.

### 3.5. Error Analysis

**Figure 2:**
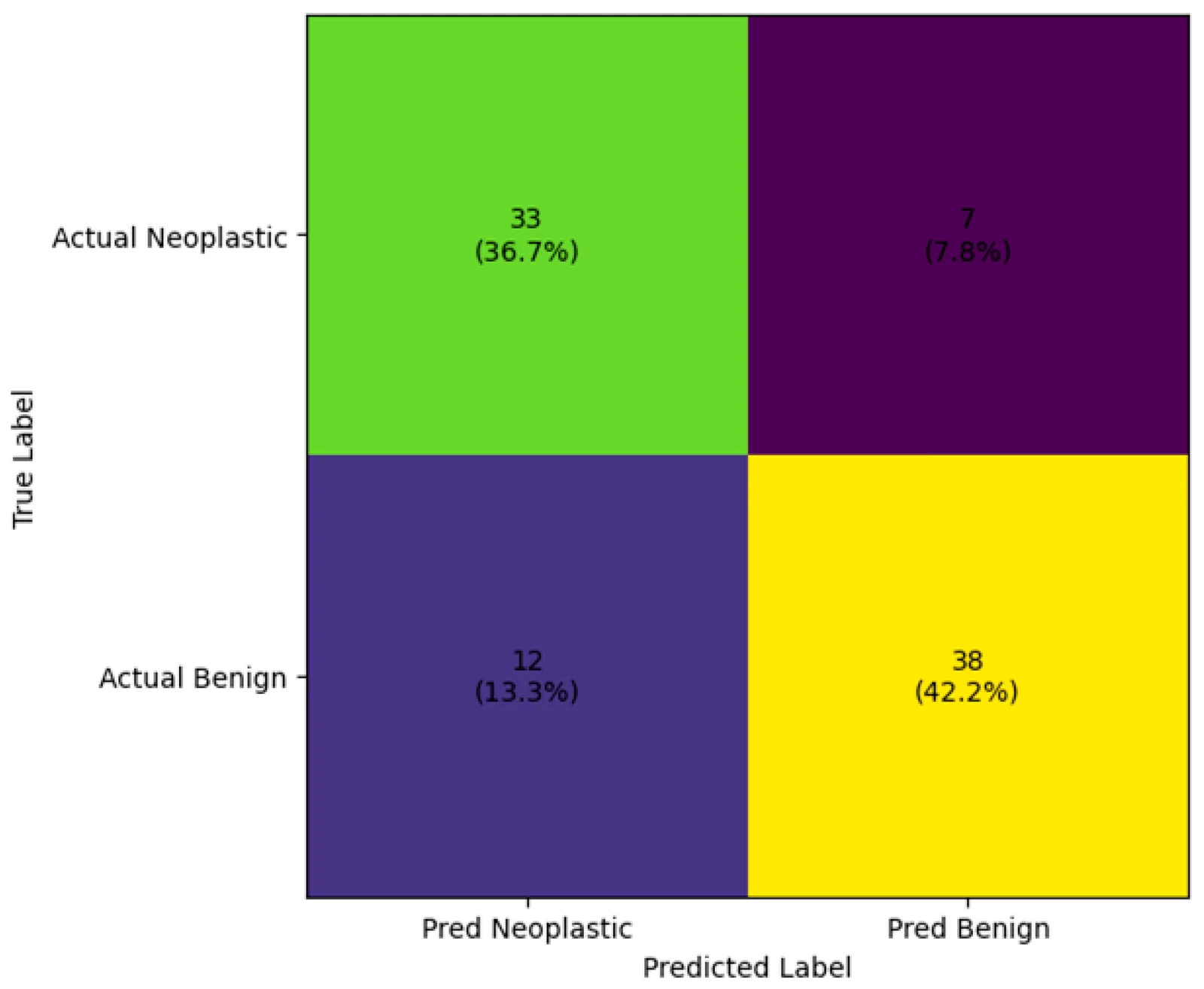
Confusion matrix of multimodal AI classification relative to histopathology. Among 40 histopathologically confirmed OSSN lesions, 33 were classified as neoplastic and 7 as benign; among 50 benign lesions, 38 were classified as benign and 12 as neoplastic, corresponding to a sensitivity of 82.5% and a specificity of 76.0%.

Misclassifications predominantly involved benign lesions with overlapping inflammatory or fibrovascular characteristics, which were more frequently classified as neoplastic. Twelve of 50 benign lesions were classified as neoplastic, corresponding to a false-positive rate of 24.0%. Conversely, 7 of 40 OSSN lesions were classified as benign, corresponding to a false-negative rate of 17.5%. Available clinical and morphological characteristics of misclassified lesions were retrospectively reviewed where retrievable from the original clinical records. Because these variables were not uniformly documented across all cases, they were used only for qualitative interpretation of error patterns and were not subjected to formal subgroup analysis. Classification performance and error distribution are illustrated in the confusion matrix (Figure 2).

### 3.6. Agreement with Reference Standard and Management Recommendations

Agreement between multimodal AI classification and clinical expert consensus was moderate (Cohen’s κ = 0.58; 95% CI, 0.42–0.74). Management recommendations were recorded as part of the original model outputs; however, case-level recommendation data required for a reproducible quantitative variability analysis were not available for retrospective reanalysis (see Section 2.5 for the reasons why case-level outputs were not retained). Management-related observations are therefore considered descriptive and no quantitative claim regarding recommendation consistency is made.

## 4. Discussion

In this exploratory single-center study, a proprietary multimodal vision-language model demonstrated measurable diagnostic performance in differentiating selected OSSN lesions from benign ocular surface lesions. In the combined multimodal condition, diagnostic accuracy was 78.9%, with a sensitivity of 82.5% and specificity of 76.0%. Accuracy was lower with expert-structured text alone (60.0%; sensitivity 60.0%, specificity 60.0%) and with images alone (70.0%; sensitivity 70.0%, specificity 70.0%), and the number of missed OSSN lesions was lowest in the multimodal condition (7 vs. 16 and 12, respectively). Although descriptive performance therefore appeared to vary according to input condition, formal claims of superiority between conditions cannot be made because case-level paired predictions required for appropriate paired statistical analysis were not retained.

Accurate identification of malignant ocular surface lesions remains essential for timely management and prevention of disease progression [1,2]. However, the clinical distinction between neoplastic and benign entities is not always straightforward, particularly in early or atypical presentations where morphological overlap may occur [24]. Although adjunctive imaging modalities such as AS-OCT and in vivo confocal microscopy have enhanced lesion characterization, diagnostic uncertainty persists in a subset of cases [25]. The present findings support the concept that artificial intelligence systems capable of simultaneously processing multiple data types may assist clinicians in navigating complex diagnostic scenarios [11]. Compared with our previous text-only evaluation of ChatGPT-4, which reported per-category accuracies of 70–85% for CIN, pterygium, and pseudopterygium [21], the present multimodal condition yielded a sensitivity of 82.5% for OSSN across a broader diagnostic spectrum that included invasive SCC and conjunctival papilloma. Differences in case mix, model version, and outcome definitions, however, preclude direct comparison between the two studies. The two cohorts, moreover, did not overlap: none of the lesions evaluated here had been included in the earlier study.

Descriptively, fewer missed tumors were observed under the multimodal evaluation condition than under the single-modality conditions. However, given the absence of case-level paired inferential analysis, this observation should be interpreted cautiously and should not be considered evidence of superiority of the multimodal approach. From a clinical perspective, minimizing false-negative diagnoses is of particular importance in oncologic settings, where delayed recognition may adversely affect prognosis. While an overly sensitive system may increase the likelihood of additional diagnostic procedures, such a profile is generally preferable to under-detection when malignancy is a concern. In this context, the observed diagnostic behavior suggests a potentially supportive role for artificial intelligence in reinforcing clinical vigilance rather than replacing specialist judgment.

Image-only analysis demonstrated greater variability in inflammatory and reactive lesions, likely reflecting the intrinsic limitations of visual interpretation when contextual clinical information is unavailable. Conversely, the lowest sensitivity was descriptively observed under the text-only condition, in which 16 of 40 OSSN lesions were missed, compared with 12 under the image-only and 7 under the multimodal condition. Given the information overlap between conditions and the absence of paired inferential comparisons, these findings should be interpreted as differences observed across input representations rather than as evidence of the independent incremental diagnostic contribution of imaging.

Recent advances in artificial intelligence have largely focused on image-based classification tasks, often relying on trained convolutional neural networks [26], including dedicated models for ocular surface tumors [12,13]. The most advanced of these, a domain-specific pretrained model built on approximately 0.76 million ocular surface images from ten centers and fine-tuned on histopathologically labeled photographs, achieved AUC values of 0.89–0.99 across internal, external, and prospective test sets, performed comparably to senior ophthalmologists in a reader study, and improved the accuracy of junior ophthalmologists when used as an aid [14]. Such purpose-built systems set a benchmark that a general-purpose model used without any task-specific training, as in the present study, should not be expected to match; the multimodal accuracy of 78.9% observed here is accordingly lower than the discrimination reported for dedicated models, although the tasks, lesion spectra, and output formats differ substantially. The potential advantage of general-purpose vision-language models lies instead in their ability to integrate heterogeneous clinical and imaging information without task-specific training and in their immediate availability. While these approaches have yielded promising results, they do not fully replicate real-world diagnostic workflows, in which clinicians synthesize imaging findings with patient history, risk factors, and examination features [11]. Similarly, benchmarking studies of general-purpose multimodal models on isolated ophthalmic images have reported limited image-interpretation accuracy in the absence of clinical context [15,16]. Reasoning-oriented large language models, which generate explicit intermediate reasoning steps, have recently achieved accuracies of approximately 90% in ophthalmic question answering, with reasoning traces that resemble clinical thinking [18]; whether such reasoning capabilities transfer to image-based lesion classification remains untested, and the model evaluated here was a non-reasoning model. Future evaluations of ocular surface lesions should therefore include reasoning-enabled multimodal models and repeated runs to quantify output stability. The present study expands upon prior work by evaluating a vision-language model designed to interpret combined clinical and imaging inputs, thereby enabling evaluation of combined clinical and imaging information within a standardized experimental framework. However, such standardized case presentation should not be considered equivalent to real-world clinical decision-making. Importantly, the objective of this study was not to evaluate artificial intelligence as a replacement for clinical expertise but rather to explore its potential as a supportive tool in diagnostically challenging situations. If prospectively validated, such systems might ultimately be explored as decision-support tools for general ophthalmologists or clinicians practicing in settings with limited access to ocular-surface oncology expertise, potentially supporting decisions regarding specialist referral. The present results do not support use of the model as a substitute for specialist evaluation or histopathological diagnosis.

Several limitations should be considered when interpreting these findings. First, the retrospective design may introduce selection bias despite efforts to include consecutive cases and a spectrum of diagnostic complexity [22,27]. Moreover, the study was conducted at a single tertiary referral center using an intentionally enriched cohort to ensure adequate representation of neoplastic lesions. Consequently, both disease prevalence and the diagnostic spectrum may differ from those encountered in unselected general ophthalmic practice, limiting the generalizability of the findings. The diagnostic spectrum was also restricted to five predefined categories and excluded other ocular surface tumors and clinically relevant mimickers, potentially simplifying the classification task.

Second, an important methodological limitation concerns the construction of the expert-structured text input. AS-OCT and IVCM findings were represented through expert-derived textual descriptors, meaning that imaging-derived information was already partially encoded in the text condition. Consequently, the text-based and multimodal conditions were not fully information-independent, resulting in potential information duplication and incorporation bias. Comparisons across input conditions should therefore be interpreted as comparisons between different representations and combinations of diagnostic information rather than as direct estimates of the independent incremental contribution of imaging. Although scenario authors and consensus raters were blinded to histopathology, the scenarios were written by ocular surface specialists, and the quality and completeness of the expert descriptors may exceed those of routine clinical documentation. In addition, case-level paired predictions were not retained for paired statistical analyses across input conditions (Section 2.5); therefore, between-condition differences are reported descriptively and should not be interpreted as evidence of statistical superiority.

Third, the evaluated system is a stochastic and proprietary generative AI model. Identical inputs may not invariably generate identical outputs, and model behavior may change following provider-side updates outside investigator control [23,27]. Repeated independent evaluations of each identical case-input combination were not incorporated into the original study protocol; therefore, run-to-run reproducibility, diagnosis switching, and management-recommendation variability could not be retrospectively quantified. Consequently, the present study provides no evidence on the reproducibility of the model’s outputs, and no claim regarding reproducibility is made. This limits assessment of model stability and should be specifically addressed in future prospective evaluations.

Fourth, individual clinician predictions were not prospectively collected under the same standardized experimental conditions. Although expert consensus contributed to clinical case characterization and reference assessment, it should not be interpreted as an individual clinician comparator. Consequently, direct comparisons between AI and individual ophthalmologists could not be performed, and the present findings do not establish superiority, non-inferiority, or equivalence of AI relative to clinicians.

Finally, the relatively moderate sample size limits the precision of estimates, particularly for lesion-specific and subgroup analyses. Nevertheless, emphasis was placed on the quality and completeness of the multimodal dataset rather than numerical expansion with heterogeneous or incompletely characterized cases. More importantly, the present study evaluated diagnostic performance under retrospectively standardized experimental conditions rather than clinical effectiveness, clinician–AI interaction, patient outcomes, or autonomous real-world decision-making. Furthermore, model performance may have been influenced by prompt design, standardized scenario construction, predefined diagnostic options, and the structured presentation of clinical information; performance under these experimental conditions may therefore differ from that observed with unstructured real-world clinical inputs. Future investigations should incorporate prospective multicenter external validation across broader lesion spectra, repeated independent model evaluations to quantify output stability, prospective archiving of all model outputs under persistent case identifiers, direct comparison with individual clinicians assessing the same standardized cases, and evaluation of clinician–AI interaction within conventional clinical pathways before clinical implementation can be considered.

Overall, multimodal artificial intelligence demonstrated measurable diagnostic performance in the evaluation of selected ocular surface lesions under standardized experimental conditions. Although the multimodal condition showed descriptively favorable performance relative to the single-modality conditions, these differences cannot be interpreted as evidence of superiority. The evaluated system should therefore be considered an exploratory decision-support approach requiring further validation rather than an alternative to specialist clinical assessment or histopathological diagnosis.

## 5. Conclusions

In this retrospective, single-center, diagnostically enriched cohort, a proprietary multimodal vision-language model demonstrated measurable diagnostic performance in differentiating selected OSSN lesions from benign ocular surface lesions. In the multimodal condition, the model achieved an accuracy of 78.9%, sensitivity of 82.5%, and specificity of 76.0%, compared with accuracies of 60.0% and 70.0% with expert-structured text alone and images alone, respectively. However, the restricted lesion spectrum, information overlap between evaluation conditions, absence of repeated-run assessment, lack of an individual clinician comparator, the absence of retained case-level paired predictions, and proprietary nature of the model substantially limit interpretation and generalizability. These findings should therefore be regarded as exploratory and should not be interpreted as evidence supporting autonomous diagnosis or immediate clinical implementation. Prospective, externally validated studies using broader lesion spectra, repeated model evaluations, and direct clinician comparators are required.

## Figures and Tables

**Table 1:** Baseline characteristics of the study population (n = 90).

Characteristic	Total (n = 90)
Age, mean ± SD (years)	58.6 ± 14.2
Sex, n (%)	
Male	54 (60.0)
Female	36 (40.0)
Laterality, n (%)	
Right eye	47 (52.2)
Left eye	43 (47.8)
Lesion location, n (%)	
Nasal	52 (57.8)
Temporal	28 (31.1)
Superior/inferior	10 (11.1)
Lesion category, n (%)	
Neoplastic (OSSN spectrum)	40 (44.4)
Conjunctival intraepithelial neoplasia (CIN)	20 (22.2)
Invasive squamous cell carcinoma (SCC)	20 (22.2)
Benign	50 (55.6)
Pterygium	20 (22.2)
Pseudopterygium	20 (22.2)
Conjunctival papilloma	10 (11.1)
Risk factors, n (%)	
Chronic ultraviolet exposure	49 (54.4)
History of ocular surface inflammation	21 (23.3)
Prior ocular trauma or surgery	12 (13.3)
Immunosuppression	6 (6.7)
Clinical features, n (%)	
Vascularized lesion	68 (75.6)
Irregular surface morphology	51 (56.7)
Limbal involvement	57 (63.3)
Feeder vessels	34 (37.8)
Imaging modalities available, n (%)	
Slit-lamp photography	90 (100)
Anterior segment OCT (AS-OCT)	90 (100)
In vivo confocal microscopy (IVCM)	62 (68.9)

AS-OCT, anterior segment optical coherence tomography; CIN, conjunctival intraepithelial neoplasia; IVCM, in vivo confocal microscopy; OSSN, ocular surface squamous neoplasia; SCC, squamous cell carcinoma; SD, standard deviation.

**Table 2:** Diagnostic performance of the multimodal vision-language model relative to histopathology (n = 90).

Metric	Value, % (95% CI)
Accuracy	78.9 (69.4–86.0)
Sensitivity	82.5 (68.1–91.3)
Specificity	76.0 (62.6–85.7)
Positive predictive value	73.3 (59.0–84.0)
Negative predictive value	84.4 (71.2–92.3)
Positive likelihood ratio	3.44 (2.06–5.74)
Negative likelihood ratio	0.23 (0.12–0.46)
False-negative rate	17.5 (7/40)
False-positive rate	24.0 (12/50)
Cohen’s κ vs. expert clinical consensus	0.58 (0.42–0.74)

Likelihood ratios and Cohen’s κ are reported as unitless values. CI, confidence interval.

**Table 3:** Diagnostic performance of the vision-language model relative to histopathology under the three input conditions (n = 90).

Metric	Expert-Structured Text	Image-Only	Multimodal
Correctly classified lesions, n/N	54/90	63/90	71/90
Accuracy	60.0 (49.7–69.5)	70.0 (59.9–78.5)	78.9 (69.4–86.0)
Sensitivity	60.0 (44.6–73.7)	70.0 (54.6–81.9)	82.5 (68.1–91.3)
Specificity	60.0 (46.2–72.4)	70.0 (56.2–80.9)	76.0 (62.6–85.7)
Positive predictive value	54.5 (40.1–68.3)	65.1 (50.2–77.6)	73.3 (59.0–84.0)
Negative predictive value	65.2 (50.8–77.3)	74.5 (60.5–84.7)	84.4 (71.2–92.3)
Positive likelihood ratio	1.50 (0.98–2.29)	2.33 (1.46–3.73)	3.44 (2.06–5.74)
Negative likelihood ratio	0.67 (0.43–1.04)	0.43 (0.26–0.71)	0.23 (0.12–0.46)
False-negative rate, % (n/N)	40.0 (16/40)	30.0 (12/40)	17.5 (7/40)
False-positive rate, % (n/N)	40.0 (20/50)	30.0 (15/50)	24.0 (12/50)

Values are % (95% CI) unless otherwise indicated. Ninety-five percent CIs for proportions were calculated with the Wilson score method and for likelihood ratios with the log method; likelihood ratios are unitless. Each case–condition combination was evaluated once; because paired case-level predictions were not retained, no inferential between-condition comparison was performed. CI, confidence interval; OSSN, ocular surface squamous neoplasia.

## Data Availability

Aggregate data supporting the findings of this study, including the per-condition classification counts, are provided within the article and its Supplementary Materials. Individual-level clinical data and source ocular images are not publicly available because of patient-privacy and institutional data-governance requirements. Additional de-identified data may be made available from the corresponding author upon reasonable request, subject to institutional approval and applicable data-protection requirements.

## Supplementary Materials

The following supporting information can be downloaded at: https://www.mdpi.com/article/10.3390/diagnostics16213475/s1. Supplementary Material S1: verbatim prompts used in each input condition, evaluation dates, model version, and interface settings (TRIPOD-LLM items).

## Abbreviations

The following abbreviations are used in this manuscript:

AI	artificial intelligence
AS-OCT	anterior segment optical coherence tomography
AUC	area under the curve
CI	confidence interval
CIN	conjunctival intraepithelial neoplasia
IVCM	in vivo confocal microscopy
LR+	positive likelihood ratio
LR−	negative likelihood ratio
NPV	negative predictive value
OSSN	ocular surface squamous neoplasia
PPV	positive predictive value
ROC	receiver operating characteristic
SCC	squamous cell carcinoma
