# Supplementary material for: Diagnostic Performance of Multimodal Artificial Intelligence in Differentiating Ocular Surface Squamous Neoplasia from Benign Lesions: A Retrospective Diagnostic Accuracy Study
Source: Diagnostics (Basel). 2026 Oct 24;16(21):3475. doi: 10.3390/diagnostics16213475 (PMC13761986; doi:10.3390/diagnostics16213475)
Supplement: Supplementary file 1 [file diagnostics-16-03475-s001.zip › diagnostics-4584033-supplementary.pdf]

**Supplementary Material S1**

# Diagnostic Performance of Multimodal Artificial Intelligence in Differentiating Ocular Surface Squamous Neoplasia from Benign Lesions: A Retrospective Diagnostic Accuracy Study

Laura De Luca, Feliciana Menna, Stefano Lupo, Enzo Maria Vingolo, Matteo Mario Carlà, Antonio Baldascino, Alessandro Meduri

*Diagnostics (MDPI), manuscript diagnostics-4584033*

## S1.1. Verbatim instructions used in each input condition

All instructions were entered in English in a newly opened chat session, one case per session. The instruction was identical across cases within each condition and differed between conditions only in the description of the material provided (expert-structured text, images, or both). No system prompt, custom instruction, or follow-up prompt was used.

### Condition 1 — Expert-structured text input

*"You are assisting with a diagnostic classification task on ocular surface lesions. Below is a structured clinical description of a single patient with an ocular surface lesion, including, where available, anterior segment optical coherence tomography (AS-OCT) and in vivo confocal microscopy (IVCM) findings. Based exclusively on this information, select the single most likely diagnosis from the following five categories: (1) conjunctival intraepithelial neoplasia (CIN); (2) invasive squamous cell carcinoma (SCC); (3) pterygium; (4) pseudopterygium; (5) conjunctival papilloma. Select exactly one category. Do not propose any diagnosis outside this list and do not answer with more than one category. Then provide a brief management recommendation corresponding to the selected diagnosis. Format the answer as: "Diagnosis: [one of the five categories]. Management: [recommendation].""*

*[Structured scenario text for the case inserted here — see S1.2]*

### Condition 2 — Image-only input

*"The attached de-identified images show the ocular surface lesion of a single patient (slit-lamp photograph and, where available, AS-OCT and IVCM images). No clinical information is provided. Based exclusively on the images, select the single most likely diagnosis from the following five categories: (1) conjunctival intraepithelial neoplasia (CIN); (2) invasive squamous cell carcinoma (SCC); (3) pterygium; (4) pseudopterygium; (5) conjunctival papilloma. Select exactly one category. Do not propose any diagnosis outside this list and do not answer with more than one category. Then provide a brief management recommendation corresponding to the selected diagnosis. Format the answer as: "Diagnosis: [one of the five categories]. Management: [recommendation].""*

*[Images uploaded as attachments in the same message — see S1.3]*

### Condition 3 — Combined multimodal input

*"The attached de-identified images show the ocular surface lesion of a single patient (slit-lamp photograph and, where available, AS-OCT and IVCM images). A structured clinical description of the same patient is provided below. Based on both the images and the clinical description, select the single most likely diagnosis from the following five categories: (1) conjunctival intraepithelial neoplasia (CIN); (2) invasive squamous cell carcinoma (SCC); (3) pterygium; (4) pseudopterygium; (5) conjunctival papilloma. Select exactly one category. Do not propose any diagnosis outside this list and do not answer with more than one category. Then provide a brief management recommendation corresponding to the selected diagnosis. Format the answer as: "Diagnosis: [one of the five categories]. Management: [recommendation].""*

*[Images uploaded as attachments in the same message; structured scenario text appended below the instruction]*

**S1.2. Expert-structured scenario template**

Each scenario was written by one of three ocular surface specialists (LDL, FM, AM), blinded to the histopathological diagnosis, using the fixed template below. The sequence and wording of the domain headings were identical across cases; fields not available for a given case were reported as "not available".

| Domain | Content (standardized wording) |
|---|---|
| Demographic characteristics | Age (years); sex. |
| Relevant risk factors | Chronic ultraviolet exposure; history of ocular surface inflammation; prior ocular trauma or surgery; immunosuppression; other (specify). |
| Symptom history | Presenting symptoms and duration (e.g., foreign-body sensation, redness, visible growth, visual disturbance); rate of growth. |
| Lesion location | Laterality; quadrant (nasal, temporal, superior, inferior); limbal involvement expressed in clock hours; corneal extension. |
| Slit-lamp appearance | Lesion size (mm); elevation; colour; presence/absence of clinically visible keratin (leukoplakia); gelatinous, papilliform, or nodular morphology; pigmentation. |
| Vascular pattern | Presence/absence of feeder vessels; intralesional vascular pattern (e.g., corkscrew, hairpin, fibrovascular); conjunctival injection. |
| Surface morphology | Regular/irregular surface; papillary fronds; ulceration; surface irregularity. |
| Lesion mobility | Mobile over the sclera / fixed to underlying tissue. |
| AS-OCT findings (when available) | Expert-derived textual description: epithelial thickness; hyperreflectivity of the epithelium; abrupt transition between normal and abnormal epithelium; subepithelial/stromal involvement. |
| IVCM findings (when available) | Expert-derived textual description: cellular pleomorphism; nuclear-to-cytoplasmic ratio; hyperreflective nuclei; loss of normal epithelial architecture; inflammatory cells. |

**S1.3. Evaluation log: model version, dates, and interface settings (TRIPOD-LLM items)**

| Item | Value |
|---|---|
| Model | ChatGPT, GPT-4o (OpenAI), accessed through the official ChatGPT web interface |

| | |
|---|---|
| Model snapshot / version identifier | Not exposed by the consumer interface; the model selector displayed “GPT-4o” throughout the evaluation period |
| Decoding parameters (temperature, top-p, max tokens) | Not adjustable in the consumer interface; provider default settings applied |
| Custom instructions | No custom instructions or system prompts were used (Section S1.1). |
| Web browsing, code execution, other tools | No external tools or custom code were used, as reported in the main manuscript. |
| Session handling | One newly opened chat per case–condition combination; no conversational history carried over |
| Runs per case–condition combination | 1 (no repeated runs) |
| Order of case presentation | Randomized within each condition |
| Images uploaded (image-only and multimodal conditions) | De-identified slit-lamp photographs and AS-OCT images; IVCM images when available. |
| Output recording | Diagnosis marked as correct/incorrect against histopathology on condition-specific scoring sheets, tallied by histopathological category; chat transcripts (including management recommendations) not exported or archived |
| Fine-tuning, retrieval augmentation, custom code | None |

**S1.4. Aggregate per-condition classification counts**

Counts underlying Table 3 of the main text (reference standard: histopathology; OSSN = CIN + invasive SCC).

| | Expert-structured text | Image-only | Multimodal |
|---|---|---|---|
| OSSN classified as neoplastic (true positives) | 24 | 28 | 33 |
| OSSN classified as benign (false negatives) | 16 | 12 | 7 |
| Benign classified as benign (true negatives) | 30 | 35 | 38 |

| | | | |
|---|---|---|---|
| Benign classified as neoplastic (false positives) | 20 | 15 | 12 |
| Total | 90 | 90 | 90 |
